# Epidemiological and PK/PD cutoff values determination and PK/PD-based dose assessment of gamithromycin against *Haemophilus parasuis* in piglets

**DOI:** 10.1186/s12917-020-02300-y

**Published:** 2020-03-05

**Authors:** Yu-Feng Zhou, Ming-Xiao Bu, Ping Liu, Jian Sun, Ya-Hong Liu, Xiao-Ping Liao

**Affiliations:** 1 National Risk Assessment Laboratory for Antimicrobial Resistance of Animal Original Bacteria, College of Veterinary Medicine, South China Agricultural University, Guangzhou, China; 2 Guangdong Provincial Key Laboratory of Veterinary Pharmaceutics Development and Safety Evaluation, South China Agricultural University, Guangzhou, China; 3 Laboratory of Veterinary Pharmacology, College of Veterinary Medicine, South China Agricultural University, Guangzhou, 510642 China

**Keywords:** Cutoff, Gamithromycin, *H. Parasuis*, PK/PD, Piglet

## Abstract

**Background:**

Gamithromycin is a macrolide approved for the treatment of bovine and swine respiratory diseases. Our study aims to establish the clinical breakpoint and optimum dose regimen for gamithromycin against *Haemophilus parasuis* in piglets.

**Results:**

Gamithromycin was well absorbed and fully bioavailable (87.2–101%) after intramuscular and subcutaneous administrations. The MICs of gamithromycin for 192 clinical *H. parasuis* isolates ranged from 0.008 to 128 mg/L and the epidemiological cutoff (ECOFF) was calculated as 1.0 mg/L. A large potentiation effect of serum on in vitro susceptibility of gamithromycin was observed for *H. parasuis*, with broth/serum ratios of 8.93 for MICs and 4.46 for MBCs, respectively. The postantibiotic effects were 1.5 h (1 × MIC) and 2.4 h (4 × MIC), and the postantibiotic sub-MIC effects ranged from 2.7 to 4.3 h. Gamithromycin had rapid and concentration-dependent killing against *H. parasuis*, and the AUC_24h_/MIC ratio correlated well with ex vivo efficacy (R^2^ = 0.97). The AUC_24h_/MIC targets in serum associated with bacteriostatic, bactericidal and eradication activities were 15.8, 30.3 and 41.2, respectively. The PK/PD-based population dose prediction indicated a probability of target attainment (PTA) for the current marketed dose (6 mg/kg) of 88.9% against *H. parasuis*. The calculated gamithromycin dose for a PTA ≥ 90% was 6.55 mg/kg. Based on Monte Carlo simulations, the PK/PD cutoff (CO_PD_) was determined to be 0.25 mg/L.

**Conclusion:**

The determined cutoffs and PK/PD-based dose prediction will be of great importance in gamithromycin resistance surveillance and serve as an important step in the establishment of optimum dose regimen and clinical breakpoints.

## Background

*Haemophilus parasuis* is the etiological agent of Glässer’s disease that causes inflammatory infections such as fibrinous polyserositis, meningitis, arthritis and bronchopneumonia in weaned piglets [1]. As a commensal pathogen in porcine respiratory tract, *H. parasuis* is frequently associated with porcine reproductive and respiratory syndrome virus, porcine circovirus type 2 and *Streptococcus suis* infections, resulting in large economic losses in the swine industry worldwide [2, 3]. There are currently 15 serotypes of *H. parasuis* and serotypes 1, 5 and 10 are the most virulent causing rapid death of infected piglets [4, 5]. The vaccines currently available only provide partial protection due to the serotype diversity and this is problematic because many strains cannot be serotyped [6]. Thus, antibiotic therapy still represents the most effective strategy for controlling the spread of *H. parasuis* infections.

Gamithromycin is a macrolide of the azalide subclass that approved for the treatment of bovine and swine respiratory diseases [7, 8]. In naturally occurring bovine respiratory diseases associated with *Mannheimia haemolytica* and *Pasteurella multocida*, the area under the concentration-time curve to minimal inhibitory concentration ratio (AUC/MIC) was the pharmacokinetic/pharmacodynamic (PK/PD) index that best predicted the efficacy of gamithromycin [9]. However, PK/PD data for gamithromycin against swine respiratory pathogens including *H. parasuis* have not been pursued. In addition, previous susceptibility study for gamithromycin against *H. parasuis* resulted in MIC_50_ and MIC_90_ values of 0.25 and 0.5 (range 0.06–4) mg/L, respectively [7]. However, as the crucial interpretative criteria to categorize the results of antimicrobial susceptibility testing (AST), the clinical breakpoints (CBPs) of gamithromycin for the relevant bacterial target pathogens still remain unclear.

In this study, we described ex vivo PK/PD relationships of gamithromycin in porcine serum against *H. parasuis* and estimated the magnitude of PK/PD parameter to achieve required efficacies. Our studies were designed to (i) determine the serum matrix effect on susceptibility, post-antibiotic effect (PAE) and post-antibiotic sub-MIC effect (PA-SME), (ii) evaluate the probability of target attainment (PTA) of the current dose of gamithromycin (6.0 mg/kg) for clinically isolated *H. parasuis* strains, and (iii) establish the relevant MIC cutoff values including epidemiological cutoff value [ECOFF; synonym of wild-type cutoff (CO_WT_)] and PK/PD cutoff value (CO_PD_; named by VetCAST as the PK/PD breakpoint) to assist the selection of a CBP for gamithromycin against *H. parasuis*.

## Results

### Gamithromycin PK profiles in serum of piglets

**Fig. 1:**
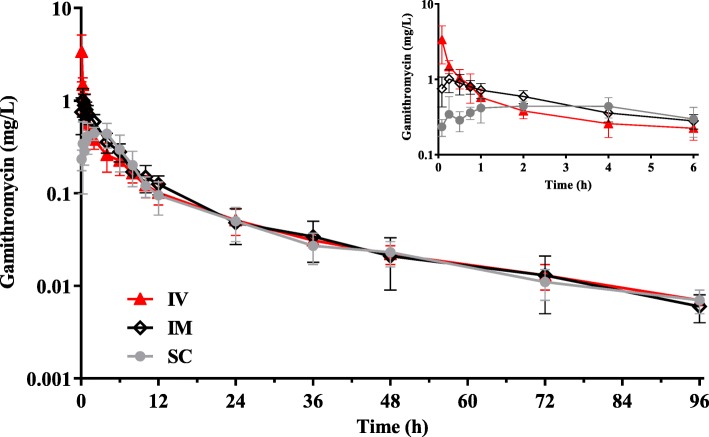
The concentration-time curves of gamithromycin in porcine serum after single IV, IM and SC administrations at 6.0 mg/kg (*n* = 6). Inset: the data plotted from 0 to 6 h

We observed gamithromycin peak levels in piglets (C_max_; 0.99 and 0.61 mg/L) at 0.36 and 1.59 h after intramuscular (IM) and subcutaneous (SC) routes, respectively (Table 1). Following a single IM injection of gamithromycin at 6.0 mg/kg, the serum drug levels were > 0.10 mg/L up to 12 h and still detectable at 96 h (Fig. 1). The serum terminal half-life (T_1/2_) ranged from 25.4 to 29.4 h after IV, IM and SC administrations of gamithromycin. Piglets received the drug IM and SC resulted in AUC_0-∞_ of 6.63 and 5.72 mg·h/L that were comparable to the IV AUC_0-∞_ at the same dose. This indicated a high bioavailability (87.2–101.1%) for SC and IM injections of gamithromycin (Table 1).

**Table 1:** PK parameters of gamithromycin in serum from six piglets following single IV, IM and SC administrations at 6.0 mg/kg (*n* = 6)

PK parameters	Unit	IV route	IM route	SC route
T_max_	h	–	0.36 ± 0.24	1.59 ± 1.40
C_max_	mg/L	–	0.99 ± 0.29	0.61 ± 0.14
T_1/2_	h	25.4 ± 2.90	29.4 ± 3.71	29.0 ± 3.44
AUC_last_	mg·h/L	6.32 ± 1.24	6.28 ± 1.56	5.45 ± 1.56
AUC_0-∞_	mg·h/L	6.56 ± 1.31	6.63 ± 1.89	5.72 ± 1.67
Cl	L/kg/h	0.92 ± 0.19	–	–
Cl/F	L/kg/h	–	0.91 ± 0.26	1.05 ± 0.30
V_ss_	L/kg	18.1 ± 3.20	–	–
F	%	–	101.1	87.2

*T*_*max*_ time to reach peak concentration (C_max_), *T*_*1/2*_ terminal half-life, *AUC* the area under the concentration-time curve from 0 to the last sampling point (AUC_last_) or from 0 to ∞ (AUC_0-∞_), *Cl* body clearance, *Cl/F* clearance scaled by bioavailability, *V*_*ss*_ volume of distribution at steady-state, *F* bioavailability

### In vitro susceptibility testing, ECOFF and PAE determinations

The calculated gamithromycin MIC for *H. parasuis* LM15 in Haemophilus test medium (HTM) broth (0.25 mg/L) was 8-fold greater than that in serum (0.031 mg/L), indicating a large potentiation effect by serum. To further confirm the serum effects, MICs and MBCs of gamithromycin were determined in both HTM and serum against 19 selected *H. parasuis* isolates. Interestingly, the geometric means of the MICs and MBCs were significantly different between HTM and serum, resulting in HTM/serum ratios of 8.93 for MICs and 4.46 for MBCs (*P* < 0.01; Table 2). For both test matrices, the MBC/MIC ratios were relatively low and ranged from 1.61 to 3.21 (Table 2).

**Table 2:** Potentiation effect of serum matrix on in vitro susceptibility of gamithromycin against 19 *H. parasuis* isolates (*n* = 19) ^*a*^

Test matrix	MIC (mg/L)	MBC (mg/L)	MBC/MIC ratio
HTM	0.52 (0.23)	0.83 (0.24)	1.61
Serum	0.06 (0.02)	0.19 (0.08)	3.21
HTM/serum ratio ^*b*^	8.93	4.46	–

^*a*^MIC and MBC represent geometric means (SD) using 19 *H. parasuis* isolates

^*b*^Comparison of Haemophilus test medium (HTM)/serum ratio differences: *P* < 0.01

**Fig. 2:**
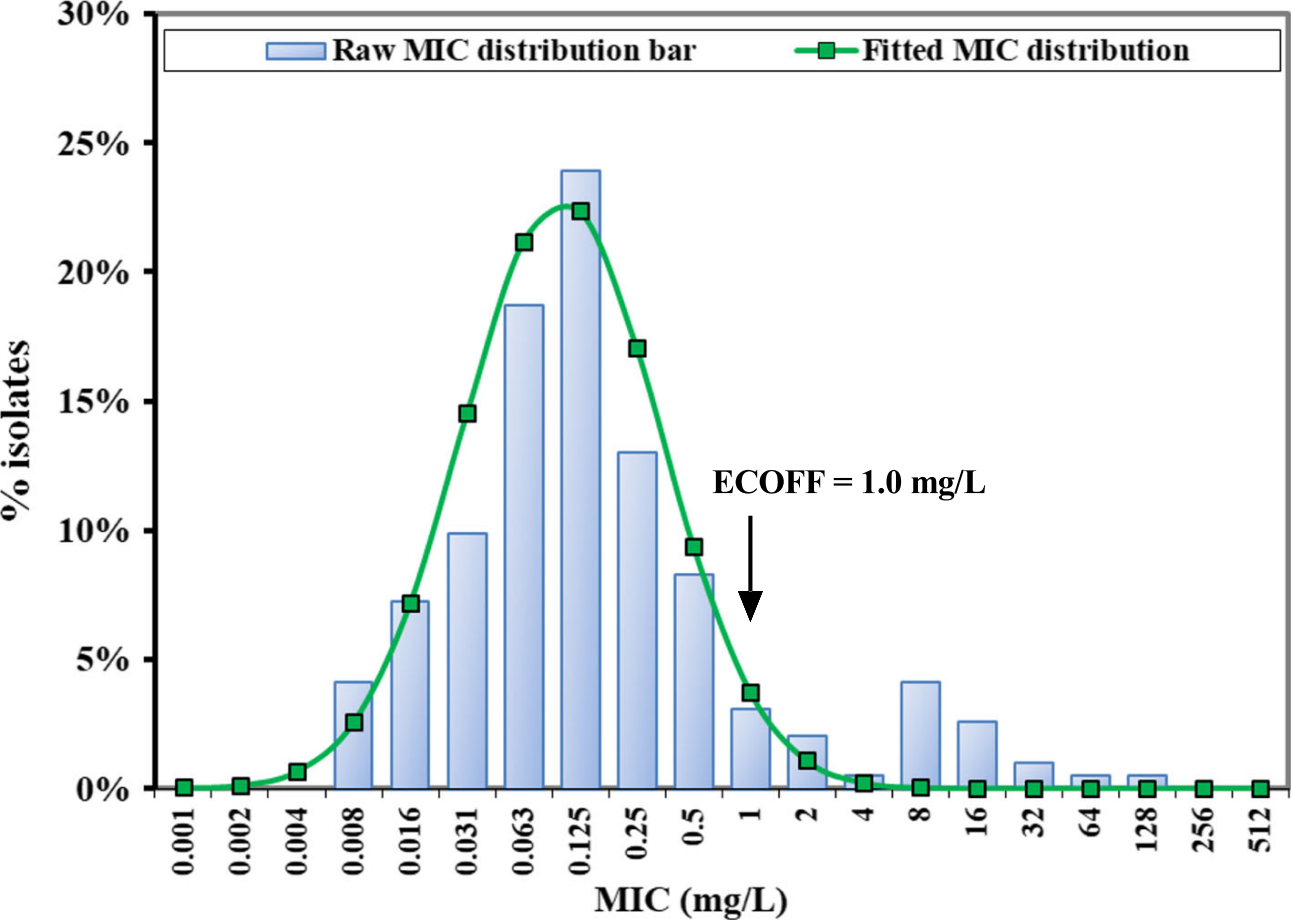
The log_2_-transformed MIC distribution of gamithromycin against clinical *H. parasuis* isolates (*n* = 192) in HTM broth. The bars represent the observed MIC frequencies, and the line represents the predicted frequency based on the best matching normal distribution [Log_2_ mean (−3.84), Log_2_ SD (1.73)]. The ECOFF value represents the epidemiological cutoff value (synonym of wild-type cutoff; CO_WT_)

The MICs of gamithromycin against our 192 clinical *H. parasuis* isolates ranged from 0.008 to 128 mg/L in HTM broth, with MIC_50_ and MIC_90_ of 0.125 and 2 mg/L, respectively. The standard goodness-of-fit tests showed that the raw MIC distribution did not match a normal distribution due to the bimodal distribution observed at the MICs of 0.125 and 8 mg/L, respectively. The 17 *H. parasuis* isolates with MICs > 8 mg/L were therefore removed to obtain the best fitted unimodal MIC distribution [Log_2_ mean (− 3.84), Log_2_ SD (1.73), *n* = 175] using the Kolmogorov-Smirnov (K-S) normality test at *P* > 0.1. The fitted MIC distribution contained > 95% that possessed gamithromycin MICs between 0.008 and 1 mg/L, and the ECOFF value was therefore calculated to be 1 mg/L (Fig. 2).

**Fig. 3:**
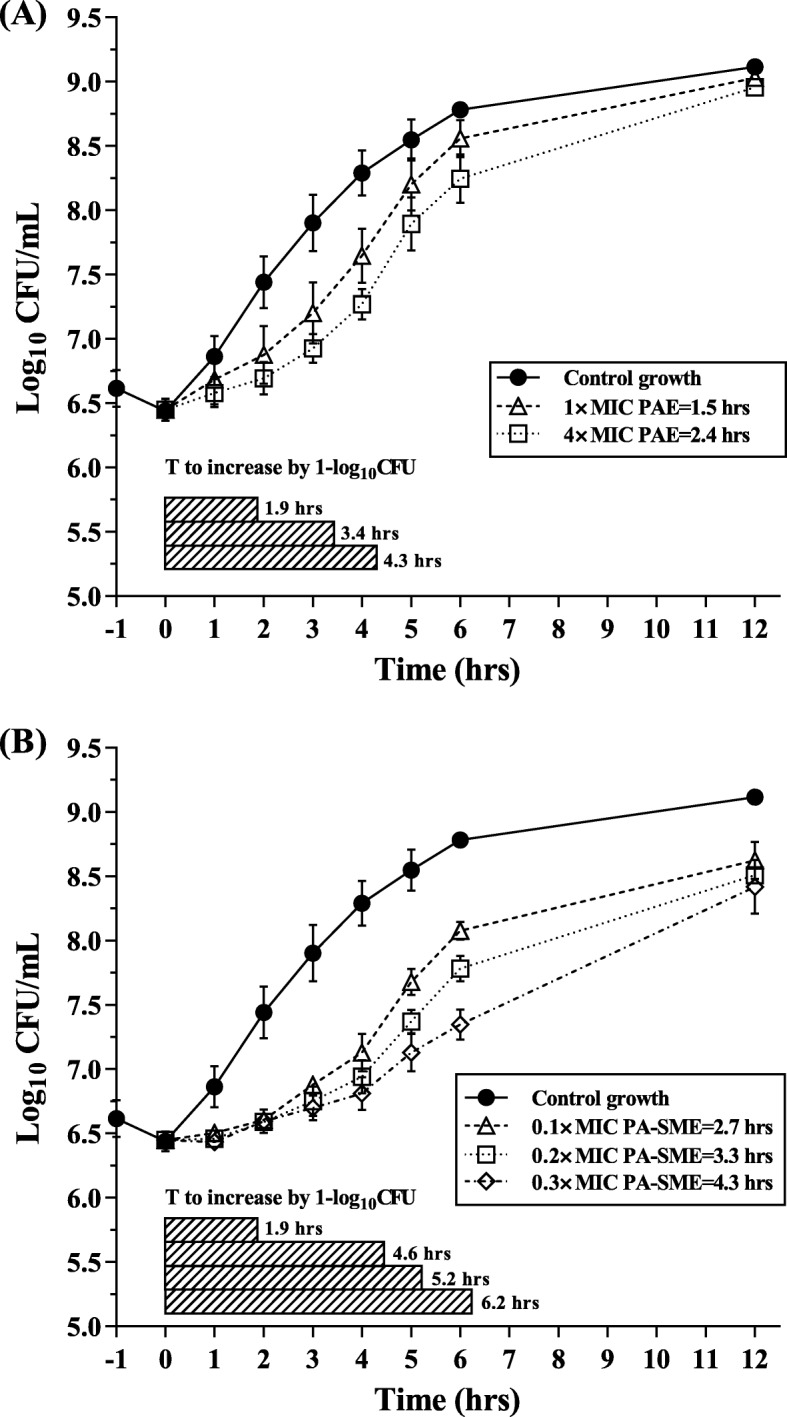
In vitro PAE (**a**) and PA-SME (**b**) values for gamithromycin against *H. parasuis* LM15. PA-SME was determined after an initial exposure to 4 × MIC. Each symbol represents the mean ± SD of data from three independent experiments (*n* = 3). The black horizontal bars represent the time required for viable counts of bacteria to increase by 1.0 log_10_cfu/mL in the drug removal and sub-MIC treatment phases

The growth of a strain of *H. parasuis* LM15 by 1.0 log_10_ cfu/mL in control group occurred at 1.9 h. After removal of bacterial cells from drug exposure at 1× and 4× MIC, regrowth was delayed to 3.4 and 4.3 h with calculated PAEs ranging from 1.5 to 2.4 h (Fig. 3a). During sub-MIC phase, as little as 0.1 to 0.3× MIC gamithromycin produced a further regrowth delay, generating PA-SMEs of 2.7 to 4.3 h (Fig. 3b).

### In vitro and ex vivo antimicrobial activities and PK/PD analysis

**Fig. 4:**
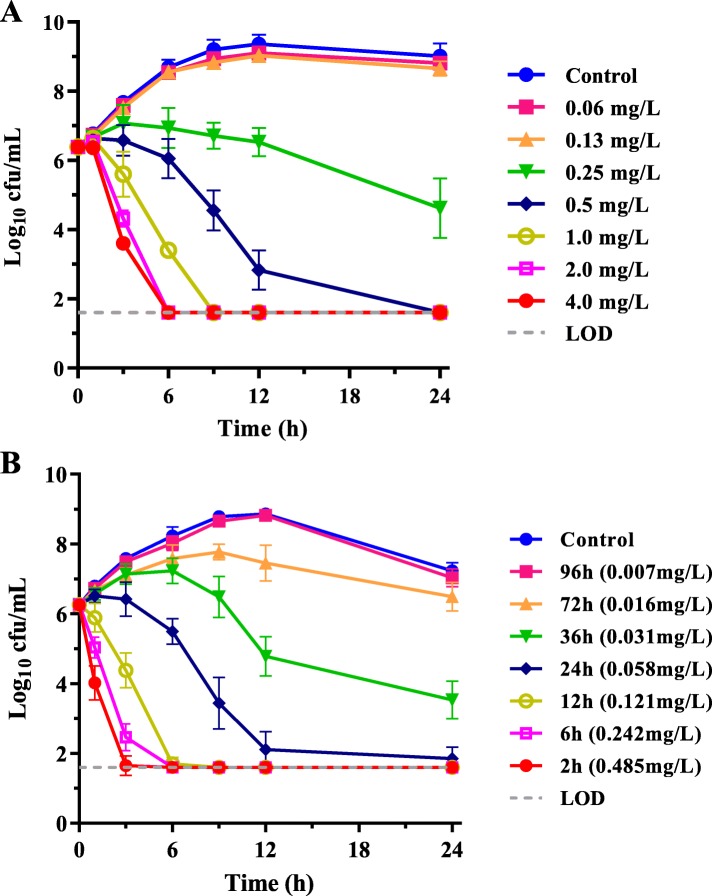
In vitro and ex vivo time-kill curves of gamithromycin against *H. parasuis* LM15 in HTM broth (MIC = 0.25 mg/L in HTM; panel **a**) and porcine serum collected before and after IM administration of gamithromycin (MIC = 0.031 mg/L in serum; panel **b**). Numerical values on right brackets in panel B are the mean concentrations of gamithromycin in serums of piglets

Gamithromycin in vitro time-kill curves using 0.25 to 16 multiples of MIC indicated a concentration-dependent killing pattern. A 0.25 mg/L drug level (i.e. MIC in HTM) produced a visible growth inhibition whereas > 3.0 log_10_ cfu/mL reductions in bacterial density occurred after exposure to gamithromycin at 2 × MIC for 24 h. At 4 × MIC or higher concentrations, *H. parasuis* densities were reduced by ~ 4.0 log_10_ cfu/mL after only 6 to 9 h exposure (Fig. 4a).

Serum collected from piglets at 36 h after IM dosing contained mean drug level of 0.031 mg/L that equivalent to MIC in serum, and exerted a visible bactericidal effect (> 2.0 log_10_ cfu/mL) after 24 h exposure. Compared with killing pattern in HTM, a more rapid and greater reduction was achieved for serums collected at 12 h and 24 h after IM administration that contained the mean drug levels of 0.121 and 0.058 mg/L (roughly 4× and 2× MIC in serum, respectively). In serum collected up to 12 h after IM injection, bacterial densities were reduced rapidly by ~ 4 log_10_ cfu/mL after 6 h exposure (Fig. 4b). There was no further regrowth demonstrating potent ex vivo bactericidal activity of gamithromycin against *H. parasuis*.

**Fig. 5:**
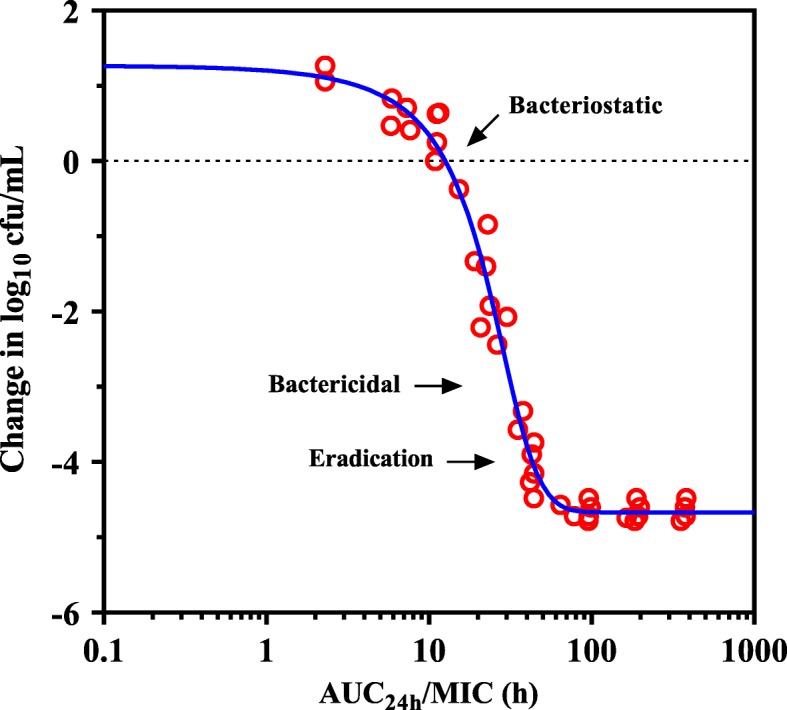
Ex vivo PK/PD relationship of gamithromycin against *H. parasuis* LM15 in serums of piglets. The curve represents predicted values based on the sigmoid E_max_ equation and the individual points represent serum samples collected at time points from 0 to 96 h

The dose-response relationship derived from ex vivo time-kill data were used to calculate the PK/PD targets in serum for typical efficacy. The AUC_24h_/MIC index correlated well with ex vivo efficacy (R^2^ = 0.97). The dose-response curve was steep, with the highest decrease from the initial density of 4.68 log_10_ cfu/mL **(**Fig. 5). The target values of AUC_24h_/MIC in serum associated with bacteriostatic, bactericidal and bacterial eradication actions were 15.8, 30.3 and 41.2, respectively (Table 3).

**Table 3:** PK/PD analysis of data acquired from ex vivo time-kill experiments of gamithromycin against a representative strain of *H. parasuis* LM15 in serums collected from each piglet (*n* = 6)

Parameter (units) ^*a*^	Mean ± SD (min to max)
E_0_ (log cfu/mL)	0.91 ± 0.13 (0.73 to 1.05)
E_max_ (log cfu/mL)	−4.68 ± 0.06 (− 4.78 to − 4.61)
E_max_ - E_0_ (log cfu/mL)	− 5.59 ± 0.15 (− 5.74 to − 5.39)
EC_50_ (h)	24.3 ± 3.88 (19.5 to 29.9)
Slope (N)	4.37 ± 2.12 (2.69 to 8.34)
AUC_24h_/MIC for bacteriostatic effect (h)	15.8 ± 4.43 (12.6 to 24.5)
AUC_24h_/MIC for bactericidal effect (h)	30.3 ± 4.40 (24.3 to 36.4)
AUC_24h_/MIC for eradication effect (h)	41.2 ± 7.48 (32.8 to 52.5)

^*a*^E_0_, the change in log_10_cfu/mL after 24 h of incubation in the no drug control sample; E_max_, difference in greatest amount of bacterial reduction (log_10_ cfu/mL); EC_50_ is the AUC_24h_/MIC producing 50% of the maximal effect; N, the slope of dose-response curve. The bacteriostatic, bactericidal and eradication effects were defined as no change, 3.0 log_10_ cfu/mL and 4.0 log_10_ cfu/mL reductions in bacterial densities

### Dose assessment and PK/PD cutoff calculation

**Fig. 6:**
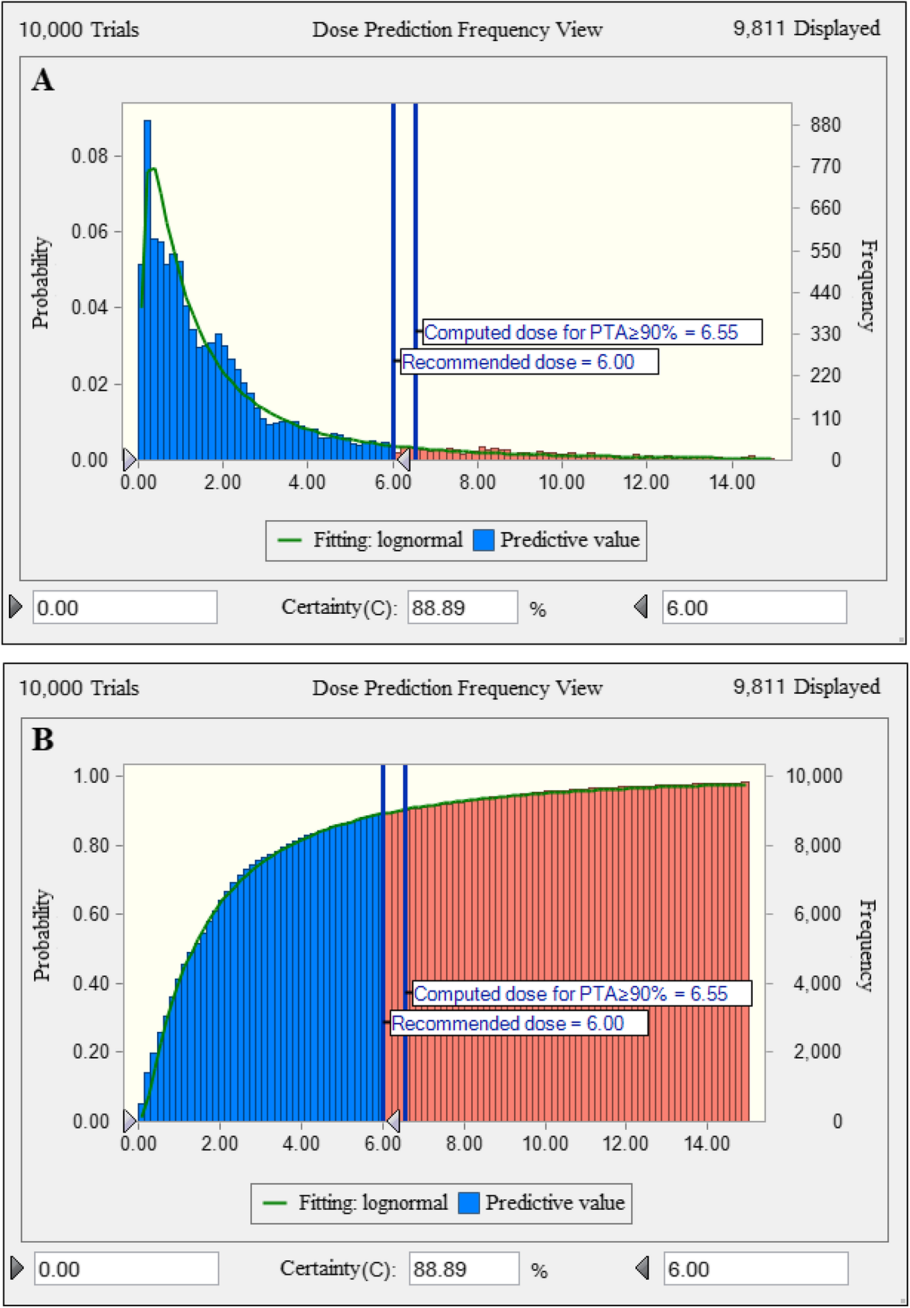
Non-cumulative (**a**) and cumulative (**b**) population distributions of gamithromycin as predicted by a PK/PD model for the treatment of *H. parasuis* infection for 3 day duration in terms of PTA (y-axis: 0–100%). The vertical bars indicate the recommended dose of 6.0 mg/kg and the computed target dose for a PTA of 90%. Dose (0 to 14 mg/kg) is indicated on the x-axis

The dose distribution of gamithromycin covering activity duration of at least 3 days needed a Cl/F of 65.5 L/kg. The scaling factor (SF) obtained by dividing the target value of AUC_24h_/MIC index by 24 h, in our case, the target AUC_24h_/MIC value for a bactericidal effect was 30.3 that equivalent to the serum concentrations over 24 h of 1.26 multiples of the MIC (i.e. SF = 1.26). The MIC distribution was divided by a scaling factor of 8.93 to bridge HTM and serum. Based on Monte Carlo simulation, the calculated dose of gamithromycin for a PTA ≥ 90% was 6.55 mg/kg. In view that the current recommended dose is 6.0 mg/kg, the corresponding PTA was calculated at 88.9% **(**Fig. 6).

Our dose prediction confirmed the adequacy of the current gamithromycin dose for *H. parasuis* infections. The PTAs of the current dose (6.0 mg/kg) for the typical AUC_24h_/MIC targets at each possible MIC in HTM were thus calculated to obtain the CO_PD_ value. At a MIC of ≤0.25 mg/L in HTM, the PTA achieving an AUC_24h_/MIC target of 30.3 (for a bactericidal effect) was 96.7% (Table 4). Therefore, the CO_PD_ value of gamithromycin for *H. parasuis* was determined to be 0.25 mg/L.

**Table 4:** The probabilities of target attainment (PTAs) for typical AUC_24h_/MIC targets at each possible MIC in HTM broth

Typical AUC_24h_/MIC targets (h) ^*a*^	PTA (%) at each possible MIC (mg/L) in HTM broth ^*b*^						
	0.063	0.125	0.25	0.5	1.0	2.0	4.0
15.8	100	100	100	95.9	31.2	0.78	0.17
30.3	100	100	96.7	37.2	1.35	0.18	0.06
41.2	100	99.5	85.4	3.31	0.57	0.12	0.05

^*a*^AUC_24h_/MIC target required to produce bacteriostatic (15.8 h), bactericidal (30.3 h) and eradication (41.2 h) effects in serum

^*b*^The scaling factor that bridged the MICs between HTM and serum has been taken into account when calculating the PTA

## Discussion

Gamithromycin is a semisynthetic triamilide antibiotic that disrupts protein synthesis by binding the ribosomal 50S subunit and causing premature detachment of polypeptide chains [7]. To our knowledge, this study is the first to address the PK/PD relationships of gamithromycin against *H. parasuis* in piglets. We demonstrated that gamithromycin was absorbed rapidly into the systemic circulation after IM and SC administrations with bioavailabilities between 87.2 and 101%. Following a single IM dose of gamithromycin at 6.0 mg/kg, serum drug concentrations remained above the MIC of *H. parasuis* LM15 beyond 36 h. Despite having limited PAE, gamithromycin has been shown to express an extended lung tissue half-life, which may also support its extended antimicrobial activity [8, 10].

Gamithromycin displayed a predominantly bactericidal activity in vitro against major swine respiratory diseases (SRD) pathogens such as *H. parasuis*, *P. multocida* and *Actinobacillus pleuropneumoniae* [7]. In studies involving field strains isolated in European areas, gamithromycin showed MIC_90_ values of 0.5, 1 and 4 mg/L against *H. parasuis*, *P. multocida* and *A. pleuropneumoniae*, respectively [8]. Similar to other veterinary macrolides such as tulathromycin, clinical field trials have indicated that gamithromycin concentrations in serum can be below the MIC_90_ for the target SRD pathogens while retaining good clinical efficacy [7, 10]. It may be due to the significant differences of AST results between serum and broth media [11, 12]. This resulted in the underestimate of gamithromycin clinical efficacy by measuring MICs in broth medium. In fact, the serum effects on antimicrobial activity of macrolides have been reported for most fastidious respiratory pathogens [11, 13]. In our study, the MICs of gamithromycin against *H. parasuis* in porcine serum were significantly lower than that in HTM. This difference in susceptibility agreed with the previous findings, in which a similar 4 to 16-fold reduction in tildipirosin MIC was observed for *A. pleuropneumoniae* by addition of 50% serum to broth medium [14]. More recently, our study regarding tulathromycin demonstrated that incorporation of increasing proportion of serum to broth from 25 to 75% progressively reduced the MIC against *S. suis* [13].

The mechanisms for increased susceptibility of *H. parasuis* to macrolides in serum matrix is likely related to the downregulation of *oprM* gene (encoding an active efflux pump) and increased outer-membrane permeability in biological fluids [15]. Supporting this notion, previous studies showed markedly lower azithromycin MICs against *Pseudomonas aeruginosa* in eukaryotic media and biological fluids such as serum and bronchoalveolar lavage fluid, compared with the recommended CA-MHB [15]. Similar observations were also noted for the potency of tulathromycin in calf serum against *M. haemolytica* and *P. multocida* [11]. This suggested that measuring MICs in eukaryotic media could be easily implemented to phenotypically detect acquired resistance to macrolides [16]. In addition, the serum effects may involve specific antibody and complement activities [11, 12]. Heat-inactivation of serum resulted in a 500-fold increase in the MIC of gamithromycin against *Mycoplasma mycoides* compared with normal serum [17]. Nevertheless, the large potentiation effect of serum would bring considerable benefit to the efficacy of gamithromycin in vivo for *H. parasuis* infections, where edema and hemorrhage from vascular leakage in affected lungs are part of the inflammatory process against respiratory diseases [14, 18]. Therefore, compared to the artificial broth that is designed to be optimal for bacteriological growth in vitro, serum is a more clinically relevant biological fluid for establishing the PK/PD relationships to predict dose regimens.

Prediction of dose distribution for gamithromycin requires selection of a robust PK/PD surrogate and determination of PK/PD targets [19]. From the rate and extent of gamithromycin killing against *H. parasuis*, a concentration-dependent killing was observed in both HTM and porcine serum, with drug concentrations of 2 × MIC being sufficient to eradicate the sample of viable bacteria. Much discussion is available in the literature surrounding the selection of the proper PK/PD index to determine the optimal dose of macrolides [9, 20, 21]. Contemporary thought on the longer acting injectable macrolides in veterinary medicine is that the large predictor of efficacy is AUC_24h_/MIC ratio [9, 22]. The AUC_24h_/MIC targets for gamithromycin against *H. parasuis* associated with bactericidal and eradication effects were 30.3 h and 41.2 h, respectively. This microbiological response in ex vivo PK/PD model was similar to or in excess of previously approved veterinary macrolides such as tulathromycin and tildipirosin [13, 23]. Further population dose prediction derived from Monte Carlo simulations indicated that the current dose of gamithromycin (6.0 mg/kg) was sufficient for treating *H. parasuis* infections, covering more than 88.9% of the MIC distribution of study clinical isolates.

CBP is used to define susceptibility and resistance. In general, the determination of CBP should take into account the ECOFF, CO_PD_ and clinical cutoff values [24, 25]. Under the clinically recommended dose, the gamithromycin CO_PD_ value (0.25 mg/L) against *H. parasuis* was lower than the ECOFF value (1.0 mg/L) in HTM broth. It probably means that the current dose (6.0 mg/kg) is a little bit too low to treat the wild-type populations. In fact, our calculated accurate dose of gamithromycin for a PTA ≥ 90% was 6.55 mg/kg in this study, although slightly larger than the current dose. However, owing to the paucity of relevant data to bridge the relationship between MIC and clinical cure, it is practically difficult to determine a clinical cutoff in veterinary medicine [24, 26]. In this case, VetCAST will not establish a CBP dividing the wild-type MIC distributions, and the ECOFF (1.0 mg/L) will therefore be recommended as surrogate [24]. This ECOFF value for gamithromycin in HTM broth is equivalent to the CLSI recommended breakpoint for erythromycin against streptococci [27]. Although the ECOFF or PK/PD cutoff could not replace the CBP, it still provides a useful interpretative criterion to categorize the AST results of gamithromycin.

## Conclusions

In conclusion, we have demonstrated that: (i) the matrix effects of serum on susceptibility is an important factor accounting for markedly augmented activity of gamithromycin in piglets and (ii) the current dose of 6.0 mg/kg gamithromycin was estimated to be appropriate, achieving bactericidal activity against *H. parasuis* with a PTA of ≥88.9%. In addition, our finding is to our knowledge the first to reveal the ECOFF and PK/PD cutoff values of gamithromycin against *H. parasuis* in piglets. However, with the paucity of clinical data for gamithromycin to establish a clinical cutoff against *H. parasuis*, the ECOFF value of 1.0 mg/L will be recommended as surrogate. Nevertheless, the PK/PD-based dose prediction and cutoff determination will provide a framework for further optimization of gamithromycin dosing regimens and for resistance surveillance.

## Methods

### Antibiotics and bacterial strains

Analytical-grade gamithromycin powder was obtained from NMT Biotech (Jiangsu, China) and reconstituted according to the manufacturer’s recommendations. Gamithromycin injectable solution (Zactran 150 mg/mL) used for PK studies was purchased commercially from Merial Animal Health Ltd., 31,000 Toulouse, France.

We isolated 192 clinical *H. parasuis* strains from heart, lung, brain, peritoneum, pericardial sac and joint fluids of diseased swine suffering polyserositis, pneumonia or meningitis in five different provinces of China in 2010 to 2018. A well-characterized representative strain of *H. parasuis* LM15 (serotype 5) was used for ex vivo time-kill experiments and PK/PD modeling because serotype 5 is the primary serotype found in China [3, 28, 29]. Species identification was performed by using MALDI-TOF mass systems (Axima-Assurance-Shimadzu) as previously described [30]. *H. parasuis* strains were cultured with Haemophilus test medium (HTM; Becton Dickinson, Sparks, MD) broth and agar containing 15 μg/mL β-NAD and 5% porcine hematin.

### Experimental design and sample collection

Six healthy castrated crossbred piglets (Duroc×Landrace×Yorkshire) weighing 14.5 to 18.6 kg (~ 1.5 to 2.0 months of age) were purchased commercially from the Guangzhou Fine Breed Swine Company (Guangzhou, China). Gamithromycin was administered intravenously (auricular vein), intramuscularly (femoral muscle) and subcutaneously (flank region) at a single dose of 6.0 mg/kg in accordance with a 3-treatment, 3-period randomized Latin square design. A 7-day washout period was allowed between administrations by each route. The piglets were maintained in accordance with the National Standards for Laboratory Animals of China (GB 14925–2010), and allowed ad libitum access to water and antibiotic-free food. The euthanasia procedure was carried out by pentobarbital sodium with intravenous injection when study was finished. All animal experiment procedures were approved by the Guangdong Association for Science and Technology [SYXK (Guangdong) 2019–0136] and the Institutional Animal Ethical Committee of South China Agricultural University (SCAU 2018A014).

Blood samples (5 mL) were collected from the jugular veins into vacutainers without anticoagulant before (0 h) and at 0.083, 0.25, 0.5, 0.75, 1, 2, 4, 6, 8, 10, 12, 24, 36, 48, 72 and 96 h after administration of gamithromycin. Serum was separated by centrifugation of blood samples at 3000×g for 10 min and stored at − 80 °C until analysis.

### Measurement of gamithromycin in serum and PK analysis

Gamithromycin concentrations in serum were determined by a validated HPLC-MS/MS method using an Agilent 1200 HPLC system linked to an API 4000 triple quadrupole mass spectrometer as previously reported [8, 31]. In brief, serum samples (0.5 mL) were mixed with 0.5 mL of acetonitrile, followed by vortex and centrifugation at 12,000×g for 10 min. The resulting supernatant was filtered through a 0.22 μm nylon syringe filter. Matrix matched calibration standards gave linear responses from 0.001 to 0.5 mg/L (R^2^ > 0.996), with limits of quantification (LOQ) of 0.0005 mg/L. All samples with drug levels > 0.5 mg/L were diluted proportionally with control serum prior to extraction with acetonitrile. Recoveries of gamithromycin from serum ranged from 95.9 to 106.2%, and both the intraday and interday variations were < 9.27% (data not shown).

All PK parameters were measured using a non-compartmental model in WinNonlin software Version 5.2.1 (Pharsight, St. Louis, MO, USA). The absolute bioavailability (F) of gamithromycin in piglets was calculated from the following equation [21, 32]:

$$ \mathrm{F}\%=\frac{{\mathrm{AUC}}_{0-\infty \mathrm{IM}\ \mathrm{or}\ \mathrm{SC}}}{{\mathrm{AUC}}_{0-\infty \mathrm{IV}}}\times 100\% $$

### In vitro susceptibility testing, ECOFF and PAE determinations

The MICs of gamithromycin against 192 clinical *H. parasuis* isolates were determined using broth dilution in accordance with CLSI guidelines [27]. *Escherichia coli* ATCC 25922 served as the quality control strain. The composition of serum and HTM broth differs significantly in electrolytes and albumin concentrations [11]. To investigate the effect of serum matrix on gamithromycin susceptibility, further MIC and MBC determinations were undertaken for 19 selected *H. parasuis* isolates in both porcine serum and HTM broth. The 19 *H. parasuis* isolates were selected to cover five different provinces of China and the whole range of gamithromycin MICs. The MBC was determined using the spot-plate technique to achieve a 3.0 log_10_ decrease in the inoculum counts [11, 13].

The epidemiological cutoff [ECOFF; synonym of wild-type cutoff (CO_WT_)] was the MIC that best describes the end of the wild-type distribution [33]. The ECOFF value for gamithromycin against *H. parasuis* was obtained using log_2_-transformed MIC distributions in HTM broth that was subjected to statistical goodness-of-fit and nonlinear least squares regression tests as previously described [34]. The mean and standard deviation (SD) of the normal distribution for optimum nonlinear least squares regression fitting of the MICs were determined using GraphPad Prism software (Version 8.0). The final ECOFF was measured as that MIC which captured at least 95% of the optimum MIC distributions using the ECOFFinder program [33].

The PAE and PA-SME were determined using a spectrophotometric method as described elsewhere [35]. The optical density was converted into bacterial counts by comparing to a standard curve (Figure S1). The PAE and PA-SME were defined as follows: PAE or PA-SME = T or T_PA_ - C, where T or T_PA_ is the time required for bacteria to increase by 1.0 log_10_cfu/mL in drug removal and sub-MIC treated phases, respectively. C is the corresponding time for untreated control [13].

### In vitro and ex vivo antimicrobial activities and PK/PD modeling

In vitro time-kill experiments of gamithromycin against *H. parasuis* LM15 were performed using HTM broth with an initial inoculum of 10^6^ cfu/mL and 0.25 to 16× MICs of gamithromycin. Ex vivo time-kill curves were established using 0.22 μm filtered porcine serum collected at specified time points from 0 to 96 h after IM dosing using a 10^6^ cfu/mL inoculum. The cultures were incubated for 1, 3, 6, 9, 12 and 24 h at 37 °C, and 10-fold serial dilutions of samples were plated on HTM agar for viable colony counts. The limit of detection (LOD) was 40 cfu/mL. The ex vivo antimicrobial effect (E) at a given gamithromycin concentration was expressed as the change in log_10_ cfu/mL after 24 h of incubation. AUC_24h_/MIC ratios were calculated at each gamithromycin concentration tested. Relationships between AUC_24h_/MIC and the ex vivo antimicrobial effect was estimated using the sigmoid E_max_ model: E = E_0_ + (E_max_ − E_0_) × C^N^ / (EC_50_^N^ + C^N^), where E_0_ is the bacterial growth in drug-free sample, C is the PK/PD index being examined (AUC_24h_/MIC), E_max_ is the greatest amount of bacterial reduction (log_10_ cfu/mL), EC_50_ is the AUC_24h_/MIC target achieving 50% of maximal effect (E_max_), and N is a sigmoid factor that describes the slope of the curve [35]. Nonlinear regression analysis was performed by WinNonlin software. The AUC_24h_/MIC targets in serum required to produce bacteriostatic (E = 0), bactericidal (E = -3) and eradication (E = -4) effect were calculated.

### Dose regimen assessment

To investigate whether the current dose of gamithromycin (6.0 mg/kg) was adequate to cover the overall MICs of *H. parasuis* population, a dose distribution prediction was simulated using Monte Carlo simulations (*n* = 10,000) with Crystal Ball software (Oracle, Redwood City. CA, USA). The dose equation was as follows [22]:

$$ \mathrm{Dose}\ \left(\mathrm{for}\ 3\ \mathrm{days}\right)=\frac{{\mathrm{Cl}}_{\mathrm{for}\ 3\ \mathrm{days}}\times \mathrm{SF}\times {\mathrm{MIC}}_{\mathrm{distribution}}\ }{fu\times \mathrm{F}} $$

Where Cl is the body clearance to cover at least 3 days (L/kg/3 day); SF is a scaling factor obtained by dividing the target value of PK/PD index, in our case, the target AUC_24h_/MIC value of 30.3 is equivalent to consider that a bactericidal effect can be obtained with a serum concentration equal to 30.3/24 = 1.26 folds the MIC (SF = 1.26 for bactericidal effect); MIC distribution in HTM broth was divided by a scaling factor of 8.93 to bridge HTM and serum; *fu* is the free drug fraction using a binding rate of 23% in porcine serum [21]; F is the bioavailability of IM dosing. In clinical practice, only susceptible *H. parasuis* isolates can be successfully treated with gamithromycin and this should be considered when calculating a dose. Therefore, for dose distribution prediction, *H. parasuis* isolates with the MICs of > ECOFF value were removed from consideration.

### PK/PD cutoff determination

A 10,000-subject Monte Carlo simulation was used to calculate the CO_PD_ value of gamithromycin against *H. parasuis* based on PK parameters, each possible MIC in HTM broth and the target AUC_24h_/MIC ratios for achieving a bactericidal effect. The AUC_24h_/MIC was calculated as follows [35]: AUC_24h_/MIC = Dose/[Cl × (MIC_HTM_/SF)]. All PK parameters were assumed to be normally distributed. SF is a scaling factor of 8.93 to bridge the MICs between HTM and serum. The CO_PD_ was defined as the MIC in HTM broth at which the PTA for bactericidal effect reached 90% [26, 29].

## Supplementary information

**Additional file 1: Figure S1.** Standard curve constructed by regression of the viable bacterial counts and optical density (OD_600nm_) of *H. parasuis* cultured in liquid medium. The solid points represent the observed data and the line represents the best fitting curve as follows: y = 0.7138 ln(x) + 10.722 (R^2^=0.9857).

## Data Availability

The datasets used and/or analyzed in this study are available from the corresponding author on reasonable request.

## Abbreviations

AST: Antimicrobial susceptibility testing
AUC/MIC: The area under the concentration-time curve to MIC ratio
CBP: Clinical breakpoint
CO_PD_: PK/PD cutoff value (named by EUCAST as the PK/PD breakpoint)
ECOFF: Epidemiological cutoff value [synonym of wild-type cutoff value (CO_WT_)]
EUCAST: European Committee on AST
MBC: Minimal bactericidal concentration
MIC: Minimal inhibitory concentration
PAE: Post-antibiotic effect
PA-SME: Post-antibiotic sub-MIC effect
PK/PD: Pharmacokinetic/pharmacodynamic
PTA: Probability of target attainment
VetCAST: Veterinary Committee for AST, a subcommittee of EUCAST
