# Supplementary material for: Epidemiological and PK/PD cutoff values determination and PK/PD-based dose assessment of gamithromycin against Haemophilus parasuis in piglets
Source: BMC Vet Res. 2020 Mar 5;16:81. doi: 10.1186/s12917-020-02300-y (PMC7059257; doi:10.1186/s12917-020-02300-y)
Supplement: Supplementary file 1 — Additional file 1: Figure S1. Standard curve constructed by regression of the viable bacterial counts and optical density (OD600nm) of H. parasuis cultured in liquid medium. The solid points represent the observed data and the line represents the best fitting curve as follows: y = 0.7138 ln(x) + 10.722 (R2=0.9857). [file 12917_2020_2300_MOESM1_ESM.pdf]

Supplementary Materials

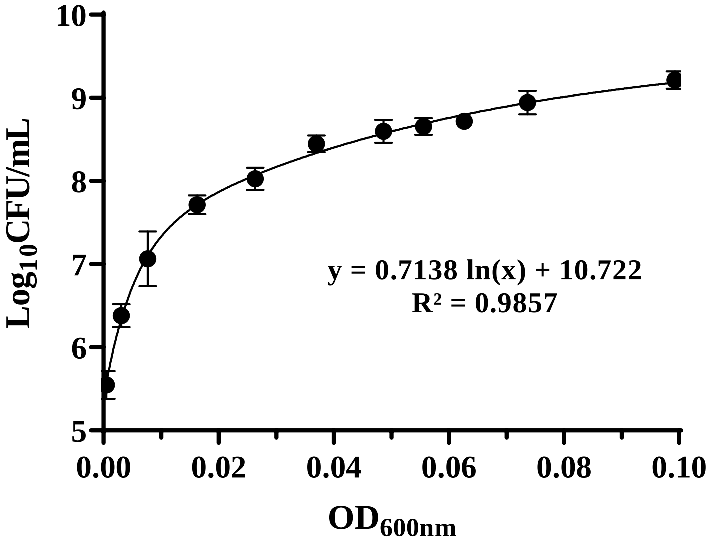

Figure S1. Standard curve constructed by regression of the viable bacterial counts and optical density (OD_{600nm}) of *H. parasuis* cultured in liquid medium. The solid points represent the observed data and the line represents the best fitting curve as follows: y = 0.7138 ln(x) + 10.722 (R^2=0.9857).
